# Coral: an integrated suite of visualizations for comparing clusterings

**DOI:** 10.1186/1471-2105-13-276

**Published:** 2012-10-29

**Authors:** Darya Filippova, Aashish Gadani, Carl Kingsford

**Affiliations:** 1 Lane Center for Computational Biology, School of Computer Science, Carnegie Mellon University, Pittsburgh, PA 15213, USA; 2 Center for Bioinformatics and Computational Biology, University of Maryland, College Park, MD, USA; 3 Department of Computer Science, University of Maryland, College Park, MD, USA

## Abstract

**Background:**

Clustering has become a standard analysis for many types of biological data (e.g interaction networks, gene expression, metagenomic abundance). In practice, it is possible to obtain a large number of contradictory clusterings by varying which clustering algorithm is used, which data attributes are considered, how algorithmic parameters are set, and which near-optimal clusterings are chosen. It is a difficult task to sift though such a large collection of varied clusterings to determine which clustering features are affected by parameter settings or are artifacts of particular algorithms and which represent meaningful patterns. Knowing which items are often clustered together helps to improve our understanding of the underlying data and to increase our confidence about generated modules.

**Results:**

We present Coral, an application for interactive exploration of large ensembles of clusterings. Coral makes all-to-all clustering comparison easy, supports exploration of individual clusterings, allows tracking modules across clusterings, and supports identification of core and peripheral items in modules. We discuss how each visual component in Coral tackles a specific question related to clustering comparison and provide examples of their use. We also show how Coral could be used to visually and quantitatively compare clusterings with a ground truth clustering.

**Conclusion:**

As a case study, we compare clusterings of a recently published protein interaction network of *Arabidopsis thaliana*. We use several popular algorithms to generate the network’s clusterings. We find that the clusterings vary significantly and that few proteins are consistently co-clustered in all clusterings. This is evidence that several clusterings should typically be considered when evaluating modules of genes, proteins, or sequences, and Coral can be used to perform a comprehensive analysis of these clustering ensembles.

## Background

Collections of protein interactions, gene expression vectors, metagenomic samples, and gene sequences containing thousands to hundreds-of-thousands of elements are now being analyzed routinely. Clustering is often used to condense such large datasets into an understandable form: it has been successfully used on protein-protein interaction (PPI) networks to discover protein complexes and predict protein function, e.g. [1]; on gene expression data to find patterns in gene regulation and essential cell processes, e.g. [2]; and on metagenomic samples to identify new species, compare them to existing clades, evaluate the diversity of a population, and suggest interdependencies between them [3,4]. In other words, clustering has become a ubiquitous part of analysis for large biological datasets.

There are many clustering algorithms available for numerical and network data, e.g. [5-12]. Each algorithm, and choice of its parameters, results in different clusterings. Sometimes, clustering algorithms must resolve ties when generating modules or may be randomized. Consequently, a single clustering algorithm may produce diverse partitions on the same data [13]. Clusterings may also change when the underlying data becomes increasingly noisy or displays variation under different conditions (such as varying gene expression levels). In addition, considering many optimal and near-optimal partitions has been shown to improve the understanding of module dynamics and the strength of relationships between individual items [14-17]. Such clusterings may offer drastically different perspectives on the data, where assessing the commonalities and differences is of great interest.

There are several ways in which the problem of diverse clusterings has been addressed. Some tools rely on a single clustering only and focus on module quality assessment, e.g. [18,19]. Comparing two or more clusterings at a time is usually done by computing a single metric, such as the Jaccard or Rand index [20], to compare clusterings side-by-side [21] or in a dendrogram [22]. These approaches can easily compare a pair of clusterings, but are not extendable to greater number of clusterings. Another approach is to aggregate multiple partitions into a consensus clustering [23,24] without delving into the differences between individual clusterings and, thus, disregarding possibly important information about the clusterings. Finally, some approaches have made steps towards visual examination of multiple clusterings: King and Grimmer [25] compare clusterings pairwise and project the space of clusterings onto a plane to visualize a clustering landscape, and Langfelder et al. [26] investigate ways to compare individual modules across multiple conditions. However, none of these approaches offer a platform for a multi-level analysis of ensembles of diverse clusterings.

In the present study, we describe Coral—a tool that allows for interactive and comprehensive comparison of multiple clusterings at different levels of abstraction. Coral allows users to progressively move from an overview to analysis of relationships between individual data items. Users may start by examining statistics on the data and individual clusterings, or by assessing the overall homogeneity of a dataset. Users can then compare any two partitions in the ensemble in greater detail. Larger scale trends become pronounced when groups of items that co-cluster often are automatically identified and highlighted. We also extend parallel sets plot [27] to show how individual items switch modules across clusterings. Coral’s visualizations are interactive and are coordinated so that users can track the same group of data items across multiple views.

## Implementation

**Figure 1:**
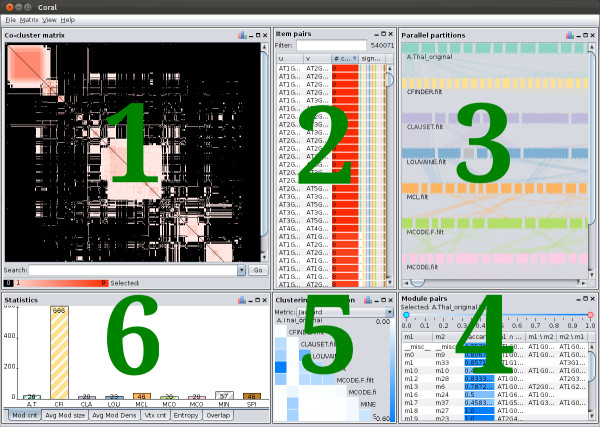
**Coral overview.** Coral views in a clockwise direction: co-cluster matrix (1), pairs table (2), parallel partitions plot (3), module-to-module table (4), ladder (5), overview statistics (6). Users may rearrange the individual views or close them to focus on fewer visualizations at a time. Data: *A. thaliana* clusterings.

In Coral’s design, we followed the visualization mantra coined by Shneiderman [28]: overview, zoom-and-filter, details-on-demand. At the overview level, Coral displays dataset statistics and highlights the most similar and dissimilar clusterings; at the mid-level, “zoomed-in,” analysis explains similarities between clusterings through module comparison; the low-level analysis compares co-clustering patterns at the level of individual data items: the genes, proteins, or sequences. The displays are coordinated [29] so selecting data in one of the views highlights the corresponding items in the other views (see Figure 1).

Coral works with modules — groups of items closely related to one another according to some metric or property. For example, modules can constitute a collection of genes that get co-expressed together or proteins forming a complex. A clustering is a collection of modules and usually is an output of a clustering algorithm. Users may also choose to group data according to attributes that come with the data such as cellular component or molecular function GO terms and use that partition as a clustering. Users may combine data from different experiemnts and across species so long as the data items that the user treats as homologous have the same IDs across the dataset.

Coral takes as an input the module files where each file represents a single clustering, and each line in the file contains a list of data items (proteins, genes, or sequence ids) from a single module, e.g. as produced by MCL, the clustering algorithm by van Dongen [5]. Coral aggregates and visualizes these data through several connected displays, each of which can be used to answer specific questions about the clusterings. Below, we examine a few such questions and describe how Coral’s visualizations help to answer them.

### How many and what size modules do clusterings have?

To gain a quick overview of their collection of clusterings, Coral users may start the analysis by reviewing basic statistics about their data: number of modules per clustering, average module size, number of items that were clustered, clustering entropy [30], and percentage of data items that ended up in the overlapping modules. Questions such as “Do clusterings have the same number of modules?” and “Are module sizes evenly distributed?” can be easily answered through these statistics. Each statistic is shown as a bar chart, and each clustering is associated with a unique color hue that is used consistently to identify the clustering throughout the system. If a clustering contains overlapping modules, the corresponding bar in the chart is striped as opposed to a solid bar for the clusterings containing no overlapping modules (see Figure 1).

### Which clusterings are the most and least similar?

**Figure 2:**
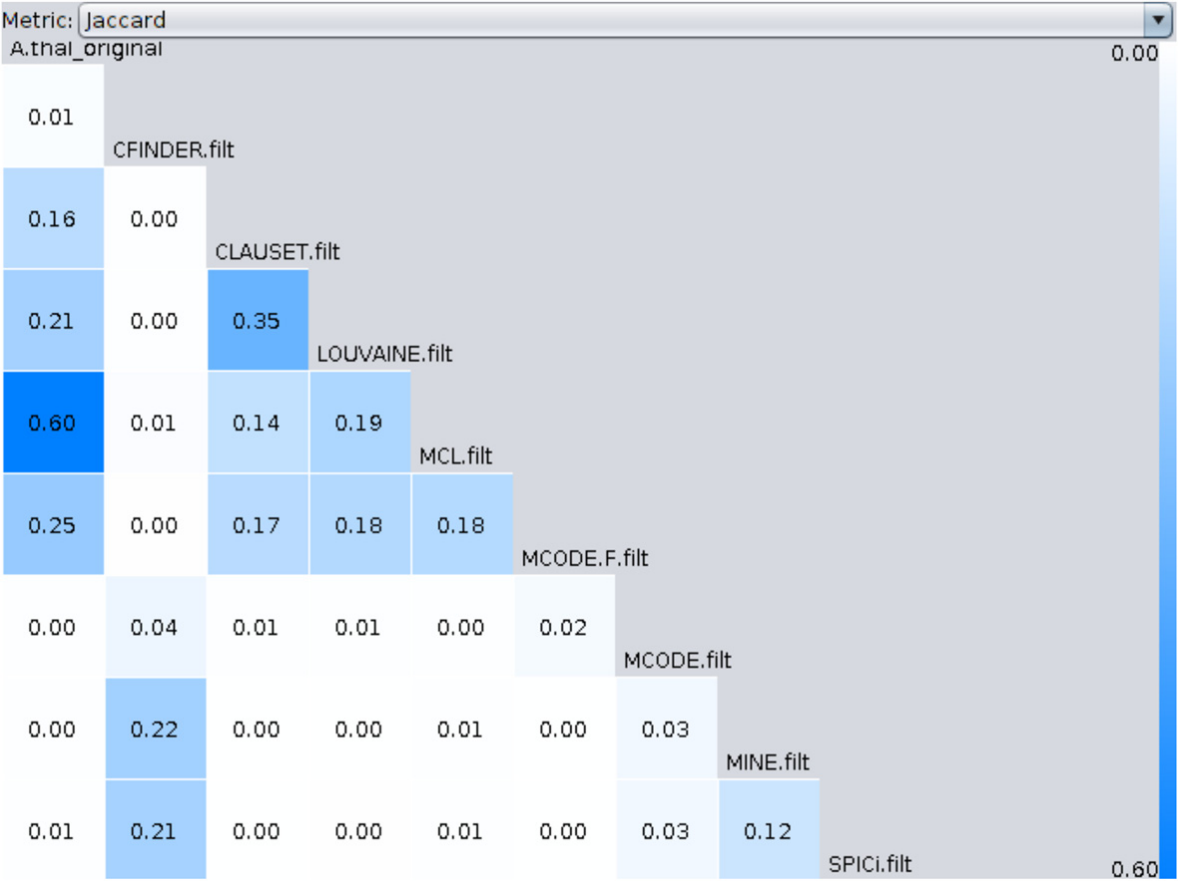
**All-to-all clustering comparison in a ladder widget.** The ladder represents a lower triangle of an all-to-all matrix where each cell (*i*,*j*) holds a score for similarity between clusterings *K*_*i *_and *K*_*j*_. Users can choose between several comparison metrics by toggling a dropdown above the ladder. Every cell is color-coded, with darker colors indicating more similarity between the pair.

Coral computes similarity scores between all clusterings and visualizes the lower triangle of the similarity matrix in a ladder widget (Figure 2). The ladder compactly displays similarity scores for every pair of clusterings in the ensemble allowing for precise comparisons. Coral offers a choice of several similarity measures to compare partitions: Jaccard coefficient, Mirkin metric, Rand index, Folkes-Mallow metric, mutual information, variation of information, purity and inverse purity, and the F-measure [30]. The ladder is color-coded as a heatmap with more intense blue cells corresponding to higher similarity scores and paler cells corresponding to low scores. Clicking a cell updates the contents of a module-to-module comparison widget (see next subsection).

### Which modules are the most similar between the two clusterings?

A natural follow-up to finding a highly similar pair of clusterings is to review the similarities between their individual modules. Is a group of interacting genes preserved between the two stages in the cell life cycle? Is there a match for a given protein complex in the PPI network of another species? Module-to-module comparison answers these questions and explains the origins of clustering similarity at a “zoomed-in” module level.

**Figure 3:**
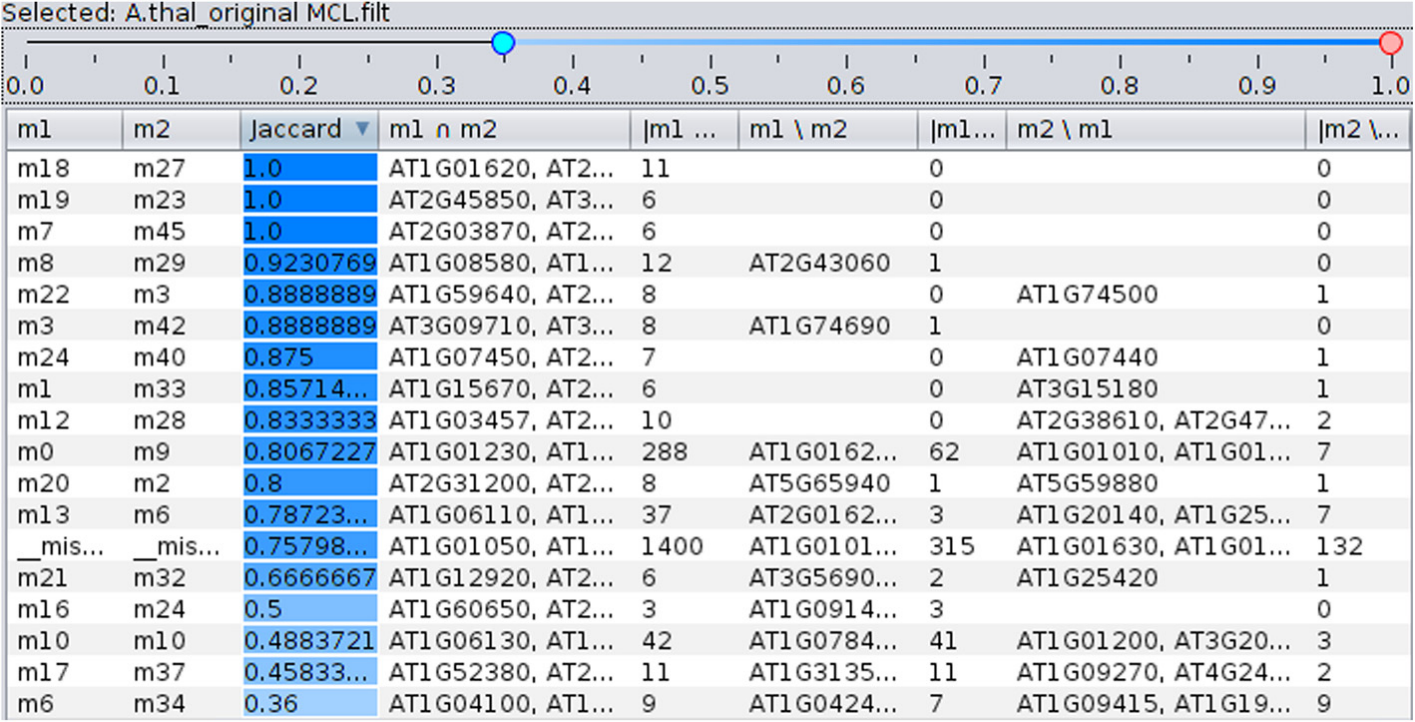
**Module-to-module comparison for two clusterings.** When users decide to focus on a pair of clusterings, they may explore all pairs of modules in a sortable table. Each module pair is shown against its Jaccard score, and lists of items in the module intersection, left and right differences. Users can filter the table rows by Jaccard score to only show rows within a given similarity range by adjusting the slider above the table. Cells holding the Jaccard scores are color-coded to indicate similarity.

For a given pair *K*_1_,*K*_2_ of clusterings, Coral calculates the Jaccard similarity $J=|m_{i}^{1}⋂m_{j}^{2}|/|m_{i}^{1}⋃m_{j}^{2}|$ between every module $\left(m_{i}^{1}\in K_{1}\right)$ and $\left(m_{j}^{2}\in K_{2}\right)$ thus capturing the amount of overlap between the two modules. For every such module pair, Coral displays the pair’s Jaccard score and items in the union, intersection, left and right set differences. All module pairs are organized in a sortable table (see Figure 3). The slider above the table allows the user to filter out module pairs for which the Jaccard score is outside the slider’s range allowing users to focus on highly similar (or dissimilar) modules. Although module-to-module analysis is possible with the parallel partitions plot (discussed below), the table offers a sortable and filterable view of the same data while supplying additional information (e.g. Jaccard index). The module-to-module table shows only the module pairs with some overlap and easily scales to hundreds of modules, thereby offering a more compact and easily navigable alternative to a confusion matrix (e.g. as used in [26]).

### Does this module appear in other clusterings?

**Figure 4:**
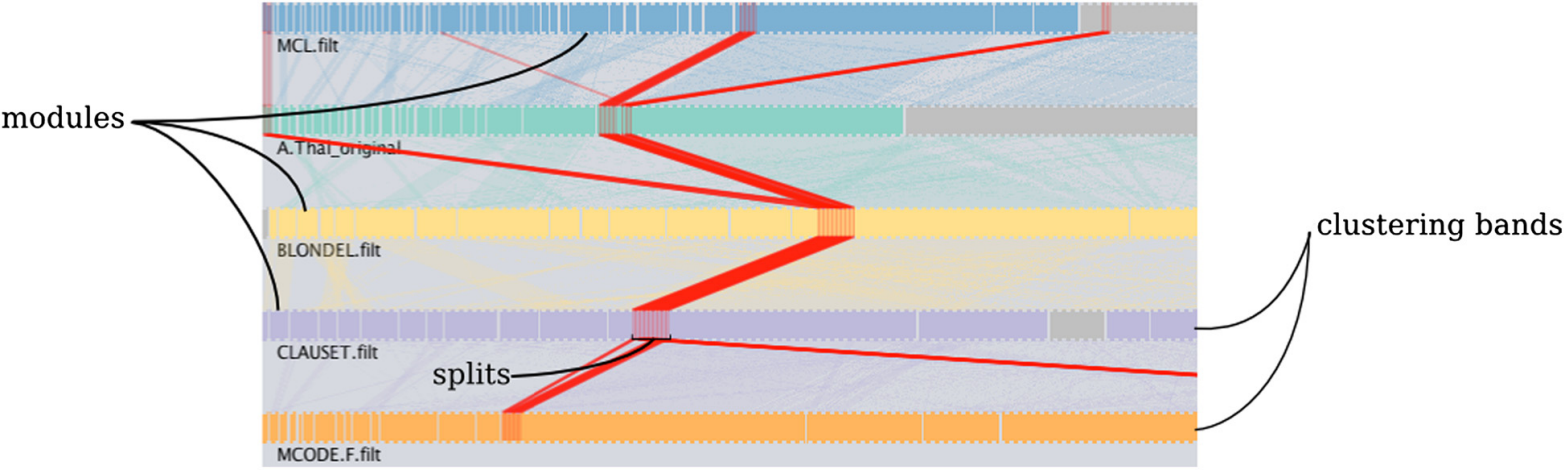
**Parallel partitions maps modules between clusterings.** Horizontal bands represent partitions, and modules are separated by blank spaces. Semi-transparent bands connect the same items from different clusterings. Red traces highlight selected items across all partitions and show how modules split and merge. Here, the user has selected a group of 215 proteins that belong to the largest core in the ensemble of clusterings for *A. thaliana* PPI network. The Louvaine, Clauset, MCL, and MCODE.F clusterings assign all of the selected proteins to a single module. For other clusterings, most of the selected proteins are placed in a grab bag region of items that were not contained in any module (shown in gray).

The ability to track individual items and whole modules across multiple clusterings provides a high level of abstraction in clustering analysis: modules may split and merge as users switch from clustering to clustering. To afford an exploration at the module level, we have developed a parallel partitions plot — an extension of a parallel sets plot used in the analysis of categorical multivariate data [27]. The parallel partitions plot represents each clustering as a horizontal band. The blocks comprising each bands represent modules, with the width of a block proportional to the number of items in that module. Semi-transparent parallelograms between clusterings connect data items with the same name. That is, each item in a clustering will be connected to its copy in the band immediately below it (see Figure 4).

The parallel partitions plot allows users to track individual items and whole modules across all partitions. To see whether a module splits or merges in other clusterings, users can select a module with a mouse while holding a shift key to highlight its members in every clustering in the plot (see red traces in Figure 4). Similarly, users may select individual items and trace them through every clustering band. The selections made in the parallel partitions plot propagate to other views making it easy to track the same group of items throughout the application. The plot is zoomable — users may zoom in to focus on a few items at a time or zoom out to see global trends across the ensemble. When the zoom level permits it, the plot displays the item labels.

The order of items in the clustering bands matches the order of items in the co-cluster matrix (discussed below) as closely as possible, while at the same time attempting to minimize the amount of crossover between the parallelograms connecting items in the consecutive clusterings. However, the items in the bands must be placed inside their respective modules. We discuss an algorithm that finds a good ordering of items in the clustering bands in the Methods section.

### What other items are in the same module as a given item *u*?

A single clustering assigns a data item *v* to a module defining its *cohort* — a set of items in the same module as *v*. Knowing the item’s module helps in assigning function to unknown proteins [6] and novel genes [2]; knowing that the item’s cohort is consistent across many clusterings increases the confidence of such predictions.

In Coral, pairwise co-cluster memberships are aggregated in a *co-cluster matrix*[24]. Given a single clustering *K*, *n *= |*K*|, we define an *n *×* n* matrix *A*^*K*^ to be *K*’s co-cluster matrix where its entries $\left(a_{\text{ij}}^{K}\right)$ are:

$$a_{\text{ij}}^{K}=\left\{\begin{array}{cc} 0\; & v_{i}\;\text{and}v_{j}\;\text{are in different modules in}\;K\\ 1\; & v_{i}\;\text{and}v_{j}\;\text{are in the same module in}\;K. \end{array}\right)$$

For some item pairs, co-clustering may be an artifact of a tie-breaking policy or a choice of an algorithm parameter: such item pairs may only co-cluster in few clusterings. On the other hand, we would like to know whether there were item pairs that co-clustered across most partitions in the ensemble. These cohort dynamics stand out if we sum up the co-cluster matrices to form a single matrix:

$$A^{+}=\overset{k}{\underset{t=1}{\sum }}A_{t}^{K},$$

where *A*^*K*^_*t*_ is a co-cluster matrix for a clustering *K*_*t*_ and *k* is the number of clusterings. Here, the $\left(a_{\text{ij}}^{+}\right)$ entries equal *k* (the number of clusterings) for item pairs (*v*_*i*_,*v*_*j*_) that have co-clustered in all partitions suggesting a strong relationship between the items, and the low $\left(a_{\text{ij}}^{+}\right)$ values correspond to pairs that co-clustered in only a few clusterings and are more likely to have been assigned to the same module by chance. The cells are colored according to their values and vary from white (low values) to red (high values). Users may zoom in and out on the matrix to focus on areas of interest.

**Figure 5:**
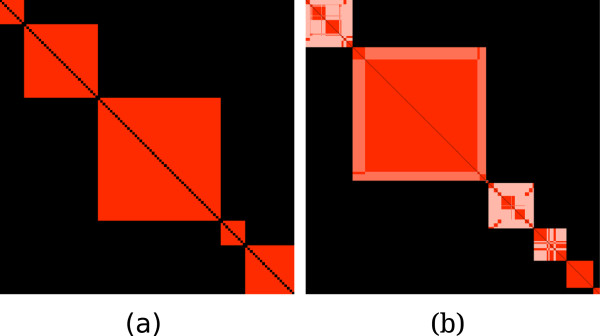
**(a) A co-cluster matrix of three identical decompositions forms blocks on the diagonal with only red cells indicating that all three clsuterings agreed (synthetic data).** (b) Big modules were broken up into smaller modules to form new clusterings (four Hybrid decompositions from the Langfelder study [16]).

**Figure 6:**
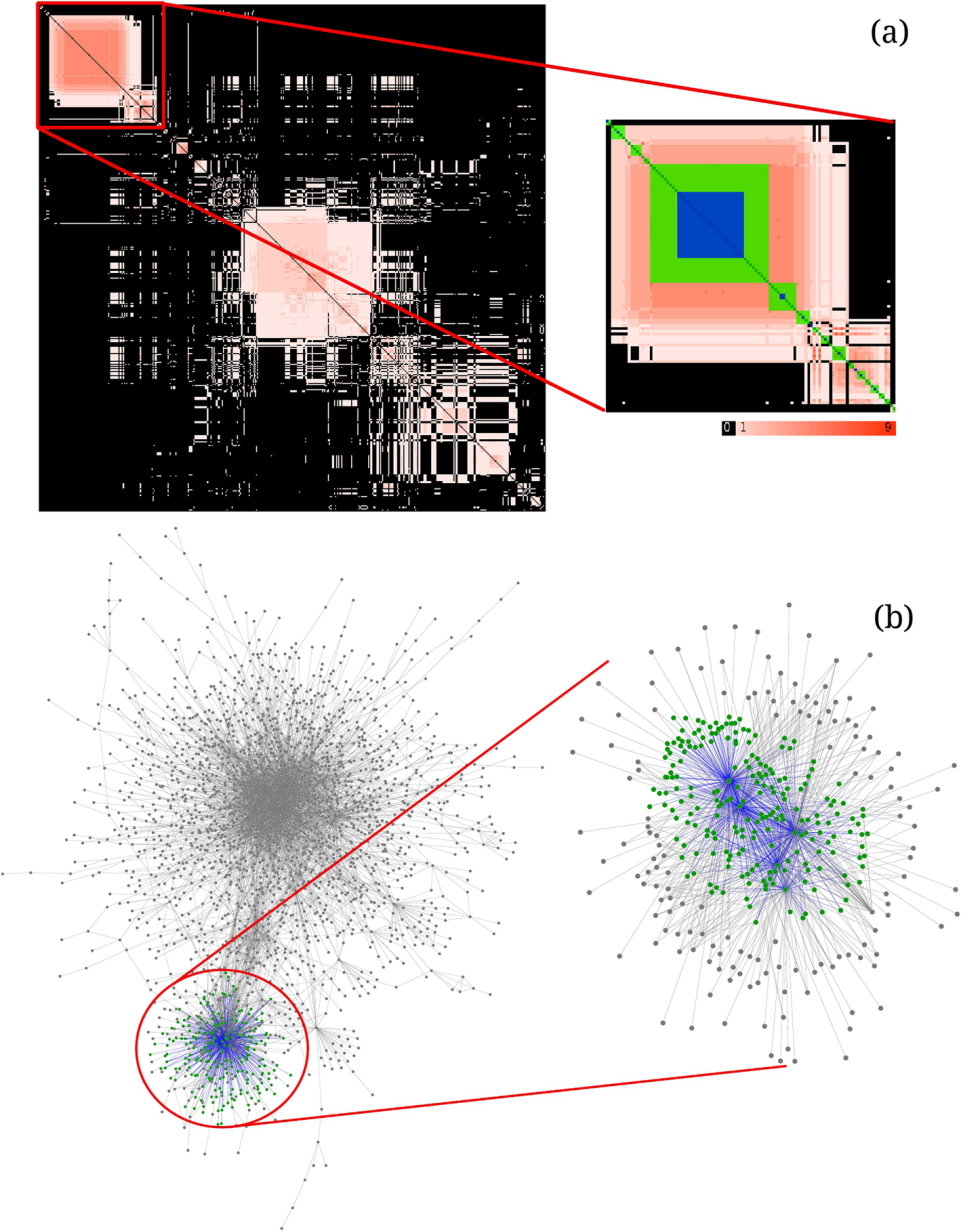
**Reordered co-cluster matrix reveals co-clustering patterns. (a)** Values in the co-cluster matrix range from 1 (light pink) to 9 (red) for nine clusterings of the *A. thaliana* PPI network with 2402 proteins from [31]. Pink regions represent item pairs that were placed in the same module by very few clusterings while regions of more saturated red represent proteins that co-clustered in most clusterings. Black indicates that the items never co-clustered. On the inset matrix, the matrix items under the green square formed a core. A large blue square overlay suggests that the core was tightly integrated into the rest of the network. **(b)** Left: nodes that formed a core in (a) are colored green, the edges between the nodes within the core are colored blue. The inset to the right shows an isolated view of the core nodes (green), edges between core nodes (blue), and nodes one hop away from the core nodes (gray). Green nodes share many edges with nodes outside of the core which resulted in the core’s low cohesion.

The co-cluster matrix is hard to read unless similar rows and columns are placed near each other. Reordering the rows and columns of *A*^+ ^brings non-zero entries closer to the diagonal and exposes any modular structure. When clusterings are highly similar, the reordered matrix will consist of blocks along the diagonal with high $\left(a_{\text{ij}}^{+}\right)$ values (Figure 5). Clusterings that are very dissimilar produce reorderings similar to Figure 6 — the diagonal blocks mostly contain low $\left(a_{\text{ij}}^{+}\right)$ values (colored white or light pink) with many entries away from the diagonal.

### Are there whole groups of items that co-cluster together often?

Groups of items that end up in the same module across many clusterings are of a particular interest because they represent the robust subclusters in the data. Such commonly co-clustered sets are called *cores*. Items in cores form the most trustworthy modules and indicate particularly strong ties between data items, increasing, for example, the confidence in protein complex identification [32] and gene annotation [33].

In a co-cluster matrix, cores translate to contiguous blocks of high-value entries. Coral finds the cores using a fast dynamic programming algorithm and highlights them within the co-cluster matrix (inset, Figure 6a). When users provide clusterings derived from a network, Coral can augment cores with an overlay showing each core’s cohesion — the ratio *E*_in_/*E*_out_ where *E*_in_ is the number of edges within the core and *E*_out _is the number of edges that have one endpoint inside the core and another endpoint outside of it [34]. When a core’s cohesion is low, the blue overlay is smaller indicating that the core shares many connections with the rest of the network (Figure 6b). Cores for which cohesion is high are more isolated from the rest of the network — these cores are distinguishable by the blue overlays that almost cover the core.

### Do items within a ground-truth clustering often co-cluster in other clusterings?

**Figure 7:**
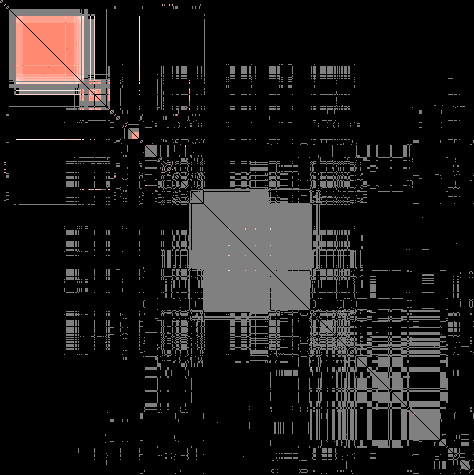
**Base clustering mode for the co-cluster matrix.** The base clustering highlights only the item pairs that co-clustered within the selected clustering graying out the rest of the matrix. Base clustering helps users focus on comparisons with the selected clustering. In this figure, the colored areas represent the original 26 *A. thaliana* modules; their mostly pink hue indicates that their item pairs co-clustered in few clusterings. Large areas of gray indicate that many novel modules found by other clusterings were not found by the link clustering algorithm [10].

When validating new protein complexes or co-expressed gene modules, users may want to see how well their results match ground-truth clusterings such as protein complexes from MIPS [35], or sets of co-regulated genes from RegulonDB [36]. In Coral, users may designate a single clustering as a *base* — a set of trusted modules with which other clusterings are expected to agree. When in this mode, Coral will only highlight those cells in the co-cluster matrix that are within the modules of the base and gray out all other non-zero matrix cells to bring users’ attention to the clustering in question. Figure 7 shows an example of a co-cluster matrix with the base set to be the *A. thaliana* modules reported in [31].

### In which clusterings do particular items co-cluster?

The co-cluster matrix displays the total number of times any two items were co-clustered, and the tooltips that appear after hovering over a matrix cell show a list of clusterings in which a given pair has been co-clustered. To facilitate sorting and search for particular item pairs, Coral provides a companion table where each row represents a pair of data items and displays the number of times the items co-clustered along with the pair’s *signature*. The signature is a *k*-long vector where the *t*^*th *^element is 1 when both data items, say, proteins, have been placed in the same module in clustering *K*_*t*_. If the pair’s items were not in the same module in *K*_*t*_, the *t*^*th *^element is set to 0.

**Figure 8:**
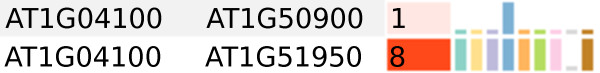
**Co-cluster signatures help track where two items have co-clustered.** Two rows from the pairs table for the *A. thaliana* dataset: each row starts with the two item IDs (here: *A. thaliana* proteins), followed by the number of times these two proteins were co-clustered, followed by a co-cluster signature that tells in which clusterings the two proteins were co-clustered. Clusterings order for this example: *A. thaliana*, Clauset, CFinder, Louvain, MCL, MCODE.F, MCODE, MINE, SPICi. Proteins AT1G04100 and AT1G51950 co-clustered in 8 clusterings. The two share many specific GO annotations: both are involved in auxin stimulus, localize to the nucleus, and participate in protein binding and sequence-specific DNA binding transcription factor activity. AT1G04100 and AT1G50900 were in the same module just once and shared no GO annotations suggesting that the relationship between these two proteins was of a different nature.

Visually, the signature’s elements that are 1 are drawn as tall solid columns and zeros are represented by the short stumps using the same color for each clustering as used in the overview statistics and in the parallel partitions plot. Figure 8 shows an example of two such pairs that have different co-cluster signatures suggesting that the relationship between the last two *A. thaliana* proteins is stronger than that of the first pair. Users can sort the rows by either the item name, the number of shared clusterings, or by the co-clustering signature. Users can also filter by the signatures to display only the rows matching a user’s pattern.

### Reordering the co-cluster matrix

The order of rows and columns in the co-cluster matrix is critical to extracting meaningful information from it. Finding an optimal matrix reordering is NP-complete for almost any interesting objective. Algorithms for the optimal linear arrangement [37] and bandwidth minimization [38] problems have been used to reorder matrices with considerable success; however, both approaches perform poorly for matrices that have many off-diagonal elements. After comparing several reordering algorithms using the bandwidth and envelope metrics, we have chosen the SPIN [39] approach that consistently produced better results on a wide range of matrices.

This approach works as follows: given a matrix *A*^+ ^, we solve a linear assignment problem (LAP) by mapping *A*^+ ^’s rows to their optimal positions in the matrix. In other words, given a bipartite graph *G *= (*R*,*I*,*E*) with *R* being the set of *A*’s rows, *I* a set of indices to which the rows will be assigned, and *E* all possible edges between nodes in *R* and *I*, we seek a matching between *R* and *I* with in a minimum cost. The edges connecting the row nodes to index nodes are weighted according to how well a row fits a particular index according to a metric that rewards rows that have non-zero entries close to diagonal and penalizes those rows that have weight away from diagonal:

$$w(i,\ell )=\overset{n}{\underset{j=1}{\sum }}a_{\text{ij}}^{+}|j-i|,$$

where *w*(*i*,*ℓ*) is the weight of assigning *i*^th^ row to *ℓ*^th^ position, and the $\left(a_{\text{ij}}^{+}\right)$ values are the co-cluster matrix entries. After permuting *A*^+ ^’s rows, the columns of *A*^+ ^must be permuted to match the row order, thus changing the weights *w*(*i*,*ℓ*) and making the row assignments found previously no longer optimal, so this process is repeated. In Coral, we use two different solvers for the LAP problem: a fast, greedy solver and the Hungarian algorithm. The greedy solver matches rows to indexes by iteratively selecting the best row-index pair; it quickly finds a starter reordering that can later be improved by the Hungarian algorithm. The Hungarian algorithm solves the linear assignment problem optimally, but because a permutation of rows invalidates the ordering of the columns, the algorithm has to be re-run for several iterations to improve the reordering. We continue iterating LAP until we get no improvement in assignment cost, observe a previously considered permutation, or exceed the maximum number of iterations.

### Identifying cores

Given a reordered co-cluster matrix *A*, we want to find contiguous areas containing high co-cluster values (*cores*). We rely on the notion of region density:

(1) $$d(p,q)=\frac{\overset{q-1}{\underset{i=p}{\sum }}\overset{q}{\underset{j=i+1}{\sum }}a_{\text{ij}}^{+}}{\left|q-p\right|}=\frac{s(p,q)}{\left|q-p\right|},$$

where a region is a square block on the matrix diagonal between rows *p* and *q*, and its density is the sum *s*(*pq*) of all matrix entries within the area divided by the area’s width |*q*−*p*|. Alternatively, we can think of the co-cluster matrix *A*^+ ^ as a weighted adjacency matrix of some graph *G*(*A*^+ ^), then *d*(*pq*) is the density of a subgraph *S* induced by the vertices *p*,…,*q*: *d*(*pq*) = |*E*(*S*)|/|*V*(*S*)|, where |*E*(*S*)| is the sum of edge weights in *S* and *V*(*S*) is a set vertices in *S*[33].

To find cores, we want to find areas on the diagonal such that the sum of their densities is highest. We do not allow the identified cores to overlap (thus we require disjoint subgraphs). We formulate the problem of finding maximally dense arrangement of areas as a dynamic program with the recurrence:

$$D_{\text{opt}}(j)=\underset{1\leq i<j}{\max}\{D_{\text{opt}}(i-1)+d(i,j)\}.$$

where *D*_opt_(*j*) is the optimal area arrangement between 0^*th*^ and *j*^*th *^item, and *D*_opt _(*n*) gives the optimal partition of a matrix *A*^+ ^into cores.

Assuming that densities *d*(*p*,*q*) are precomputed and require only a constant time to look up, the dynamic program above takes *O*(*n*^2^) time (for each *i*, we solve at most *n* subproblems, and *i* ranges from 1 to *n*). However, a brute force algorithm for computing the block sums *s*(*p*,*q*) (and, hence, the densities) in equation 1 must iterate through every pair 1 ≤* p *<* n*, *p *<* q *≤* n*, each time computing a sum of |q−p+1|2 entries, resulting in a runtime of *O*(*n*^4^). This can be improved because the sums are related. We have:

$$s(p,q+1)=s(p,q)+\overset{q}{\underset{i=p}{\sum }}a_{i,q+1},$$

making it possible to compute all *s*(*p*,*q*) in *O*(*n*^2^) time. This reduces the total runtime to find cores to *O*(*n*^2^ + *n*^2^) =* O*(*n*^2^).

The algorithm finds a series of areas of varying size and density. Some areas are of no interest and were included in the series only because every block contributes a non-zero score to the total sum. To focus on the meaningful regions only, we filter out the cores with density less than the average density. To calculate the average density for a region *p*,…,*q*, we first compute an average cell value for *A*^+ ^:

$$w_{\text{avg}}=\frac{s(1,n)}{\bar{z}},$$

where $\bar{z}$ is the number of non-zero cells in *A*^+ ^. We then define a probability of an edge existing in a graph induced by *A*^+ ^:

P(e)=z¯n−12.

Then, for a given ordering of the matrix *A*^+ ^, let *S* be a subgraph induced by vertices *p*,…,*q*. Then *h*_*pq *_= |*q*−*p* + 1| is the number of vertices in *S* and hpq2 is the maximum number of edges *S* can possibly have. For this block, the expected block density would be:

davg(p,q)=wavgP(e)hpq2hpq=s(p,q)z¯z¯n−12hpq2hpq=hpq2s(p,q)hpqn−12.

The areas that have density higher than their *d*_avg_(*p*,*q*) represent groups of data items that have co-clustered together more often than is expected by chance. Hence, Coral displays only these cores.

### Ordering in parallel partitions

When ordering clustering bands in the parallel partitions plot, we would like to put similar clusterings next to each other and avoid putting two dissimilar clusterings vertically adjacent. The intuition for such a constraint is that if the two clusterings *K*_*i *_and *K*_*i*+1 _share many similarities, the bands connecting individual items between the clusterings will only cross a few times making it easier to track module changes. We also need to order items within the bands in a way that puts items from the same module next to each other and does not allow items from other modules to interleave.

To find a vertical order for the clustering bands, we apply a greedy algorithm that uses clustering similarity scores. First, we compute the similarity for every pair of clusterings sim(*K*_*i*_,*K*_*j*_) using Jaccard. Next, we find the two most similar clusterings *K*_1_,*K*_2_, add them to a list, and look for a clustering most similar to either *K*_1_ or *K*_2 _(whichever is greater). We proceed by repeatedly picking the clustering that is most similar to the last clustering added to the list. The order in which clusterings were added to the list determines the order of the clustering bands.

We pursue two objectives when ordering items and modules within a single clustering band: items that belong to the same module must be placed next to each other, and the ordering has to be similar to the column ordering in the co-cluster matrix (so as to maintain the user’s mental mapping between the two visualizations). To preserve the matrix ordering in clustering bands, each module is best placed in a position where most of its items are close to the matrix columns corresponding to those items. However, the order of the columns in the matrix may be such that two items *u* and *v* from the same module are far apart in *A*^+ ^. We propose a heuristic to solve this ordering problem: given an ordering of the columns in the matrix *A*^+ ^, for each module *m*_*i *_in clustering $\left(K=\{m_{1},\ldots ,m_{k_{i}}\}\right)$ we compute its rank based on how “displaced” items in the module are relative to the positions of the module’s items in the matrix:

$$d(m_{j})=\underset{u\in m_{j}}{\sum }i(u),$$

where *i*(*u*) is the index of a column in *A*^+ ^corresponding to the data item *u*. Modules that should be placed first in the clustering band would have the lowest rank, so we sort the modules in order of increasing *d*, and the module’s position in the sorted array determines module’s position in the clustering band.

## Results

### Data

*Arabidopsis thaliana* is a model organism widely used in plant science, but out of its 35,000 predicted proteins one third still lack an assigned function [40]. A recent publication reports a new protein interaction network for *A. thaliana* that covers a part of the plant’s proteome not studied previously [31]. We have selected several clustering algorithms that are often used on PPI networks (Table 1) and, for each of the algorithms, we have generated a clustering of the largest connected component of the *A. thaliana*’s network. To test the resulting modules for robustness, we compare this ensemble of clusterings to the modules reported by the authors of [31] who used a link-clustering method by Ahn, Bagrow, and Lehman [10]. Prior to comparison, we filtered the newly generated modules using the same criteria as [31] by removing modules of size smaller than 6 and with partition density < 0. The new modules were tested for GO enrichment with FuncAssoc [41] (see Table 1 for details).

**Table 1:** **Clustering algorithms used on *A. thaliana* network**

**Algorithm**	**Proteins**	**Modules**	**Enriched**
Louvain[9]	2369	23	21
CFinder[8]	508	666	180
Clauset[7]	2313	20	18
MCL[5]	844	46	33
MCODE[6]	268	20	16
MCODE.F[6]	1314	20	19
MINE[12]	206	57	29
SPICi[11]	259	46	27

Statistics on clusterings of the largest connected component of the *A. thaliana* network. All algorithms were run using their default settings. MCODE was run without “haircut” and no “fluff,” MCODE.F included “fluff.” The table reports the number of proteins that were assigned to at least one module, the number of modules after filtering according to procedure used in Vidal et al. [31], and the number of modules FuncAssoc [41] reported as enriched for at least one GO annotation.

For our comparison, we have focused on the graph clustering algorithms for which the implementations were available (see Table 1). Louvain[9] and Clauset[7] are two algorithms that search for a network partition with highest modularity [42]. Both tend to find large clusters and usually cover most of the nodes in a network. CFinder[8] is a clique percolation method that identifies overlapping communities by continuously rolling cliques of an increasing size. Resulting clusterings usually contain many small modules with a high amount of overlap and cover only a part of the network ignoring graph structures like bridges and stars. MCL[5] is a fast, flow-based clustering algorithm that uses random walks to separate high-flow and low-flow parts of the graph. Its modules tend to be small and usually cover most of the input network. MCODE[6] algorithm finds modules in biological networks by expanding communities around vertices with high clustering coefficient. “Fluff” and “haircut” options for MCODE allow to add singleton nodes connected to the module by just one edge and to remove nodes weakly connected to the module correspondingly. MINE[12] is closely related to MCODE, but uses a modified weighting scheme for vertices which results in denser, possibly overlapping modules. SPICi[11] grows modules around vertices with high weighted degree by greedily adding vertices that increase module’s density. The partitions contain many dense modules, but usually cover only a part of the network.

### Applying Coral to *A. thaliana* clusterings

To get an overview of the data, we review various statistics on the generated clusterings. For the majority of the clusterings, modules that remained after filtering covered only a portion of the network. The two clusterings produced by the modularity-based methods, Louvain and Clauset, were the only clusterings that included more than 95% of all proteins into their modules. The number of modules per clustering varied significantly from 20 to 666 (Table 1). The average module size was highest for Clauset (115.65), Louvaine (103.00), and the MCODE.F (82.05) clusterings significantly exceeding the average module size among all other clusterings (3.02-26.31 items per module). For the original 26 *A. thaliana* modules [31], 3% of the proteins were assigned to more than one module; in the CFinder clustering over half of the clustered proteins (59%) participated in multiple modules.

The nine *A. thaliana* clusterings are highly dissimilar: most cells in the ladder widget (Figure 2) are white or pale blue, and the majority of pairwise Jaccard similarity scores are below 0.07. MCL yielded the partition most similar to *A. thaliana* modules reported in [31] (A.Thal original) with Jaccard similarity of 0.60. Surprisingly, the 26 modules generated by link clustering [31] shared very little similarity with CFinder, the only other algorithm in the ensemble designed to produce overlapping modules.

Low pairwise similarity scores between so many pairs of clusterings is easily explained using the module-to-module table: clusterings with Jaccard similarity below 0.07 overlap by a few small modules or no modules at all. The similarity of 0.60 between MCL and A.Thal (Figure 4) may be attributed to the two big modules that are largely replicated between the two clusterings: the module m9 from MCL and the module m0 from *A. thaliana* (highlighted row) overlap by 288 proteins with Jaccard similarity 0.8. Several smaller modules (shown at the top of the table) are duplicated exactly between the two clusterings.

The co-clustering matrix for *A. thaliana* clusterings contains several large regions of co-clustered proteins along the diagonal (Figure 6), however, most cells are pale indicating that they were co-clustered by only a few clustering algorithms; very few matrix cells are close to the saturated red. Indeed, 65.25% of all co-clustered pairs of *A. thaliana* proteins have co-clustered just once across all of the nine clusterings used in the analysis and only 6.34% of protein pairs were co-clustered in 5 or more partitions. This low number of protein pairs that were assigned to the same cluster means that the clusterings in the ensemble mostly disagreed.

The dynamic program for identifying cores found 249 areas in the *A. thaliana* network in which proteins co-clustered more often than could be expected by chance, with the largest core containing 215 proteins and with the average number of proteins per core of 10.38 proteins. Most cores, including the largest core, had low cohesion values indicating that the proteins forming the cores had many connections to proteins outside of the cores (see Figure 6). This finding is correlated with the fact that the clusterings did not agree in general and only small sets of proteins were consistently clustered together across the ensemble.

Finally, setting A.thal original to be the base clustering shows that the modules found by [31] covered only a fraction of modules found by other methods, although they included the largest core. The majority of A.thal original modules were colored pale pink indicating that modules found by the link clustering were found by no more than 3 other clustering methods. We trace the largest core in the parallel partitions plot (Figure 4): the proteins in the core are co-clustered by A.thal original, Clauset, Louvaine, MCL, and MCODE.F while SPICi, MINE, and MCODE ignored the majority of core’s proteins completely. CFinder, with its many overlapping modules of size 3, 4, and 5, clusters some of the core’s proteins and puts a large part of the core in the grab bag group representing unclustered proteins.

## Discussion

Clustering algorithms may generate wildly varying clusterings for the same data: algorithms optimize for different objectives, may use different tie breaking techniques, or only cluster part of the data items. A popular technique for optimizing modularity has been shown to suffer from a resolution limit [43] and multiple decompositions may have the same modularity value [14]. When a true decomposition is available, the clustering quality can be quantified using the similarity score and the true positive and true negative rates. However, when there is no true clustering, it is hard to decide which clustering is better than the others. We propose that researchers generate several clusterings by either using different clustering algorithms or varying algorithm parameters. Coral can help compare such clusterings and identify cores in which the data items co-cluster across multiple clusterings.

Most views and statistics in Coral work for both non-overlapping and overlapping clusterings. All overview statistics extrapolate well for overlapping modules except for entropy which assumes that no two modules overlap and therefore may overestimate the actual entropy. The co-cluster matrix naturally adapts to overlapping modules by allowing their corresponding blocks to overlap. Currently, if a pair of data items co-occur in more than one module within a single clustering, their co-cluster value is set to 1 and is not weighted higher relative to other pairs. The parallel partitions plot assumes that the modules in individual clusterings do not overlap. However, if there are overlapping modules, parallel partitions will still lay out the modules in a line by duplicating the co-occurring element in every module in which it occurs.

Although the examples we use in this paper are based on network clustering, Coral does not require its input data to be a network partition and can be used with equal success on gene expression or classification data. In particular, if users would like to compare several classification results, they can do so in the same manner as we have demonstrated for the *A. thaliana* clusterings. The similarity measures of purity, inverse purity, and the F-measure implemented in Coral are helpful in comparing classifications to the available truth. The module-to-module table is a more flexible alternative to the confusion matrix that is often used to evaluate classification results.

Coral has been used to analyze clusterings of up to 4115 items. The runtime varies with the number of clusterings, number of modules and data items per clustering, and the size of the modules. The startup operations — parsing the input clusterings, computing dataset statistics and all-to-all clustering similarities, as well as rendering the views — take from under a second to 11 seconds for clusterings from 33 to 4115 data items. Matrix reordering is the single biggest performance bottleneck for Coral. Reordering the co-cluster matrix for 2376 *A. thaliana* proteins took, on average, 29 seconds when to reorder using the greedy heuristic and 70 seconds when to reorder using the Hungarian algorithm. However, both the greedy heuristic and the Hungarian algorithm find good orderings after very few iterations and the reordering only needs to be computed once before analysis. Solutions for LAP computed with the Hungarian algorithm improve with every iteration and usually converge on a good reordering fast.

## Conclusions

Coral offers a comprehensive array of visualizations that allow users to investigate modules from various viewpoints inlcuding several novel views. Coral guides users from overview statistics implemented as familiar bar charts to detailed cluster-to-cluster comparison in a table. The ladder widget, a lower triangle of the comparison matrix, helps users pick the most similar (or dissimilar) pair of clusterings and to judge how similar clusterings in the dataset are overall. A color-coded co-cluster matrix shows how often any pair of items in the dataset have been placed in a module together. A novel adaptation of parallel coordinates, parallel partitions plot, makes tracking a group of items across clusterings easy with intuitive selection techniques. These views combined create a powerful tool for a comprehensive exploration of an ensemble of clusterings. Coral can help users generate questions and hypotheses about the data that could be later definitively answered with the help of additional experiments.

## Availability and requirements

- **Project name:** Coral

- **Project home page:**http://cbcb.umd.edu/kingsford-group/coral/

- **Operating systems:** platform-independent

- **Programming language:** Java

- **Other requirements:** Java 1.6 and 1Gb of RAM

- **License:** GNU GPL

## Competing interests

The authors declare that they have no competing interests.

## Authors’ contributions

CK and DF conceived the project, DF is the designer and developer for the application. CK provided design considerations and worked on developing some of the algorithms. AG implemented reordering algorithms for co-cluster matrix. DF and CK contributed to final manuscript. All authors read and approved the final manuscript.
